# Glycative Stress, Glycated Hemoglobin, and Atherogenic Dyslipidemia in Patients with Hyperlipidemia

**DOI:** 10.3390/cells12040640

**Published:** 2023-02-16

**Authors:** Chien-An Yao, Tsung-Yi Yen, Sandy Huey-Jen Hsu, Ta-Chen Su

**Affiliations:** 1 Department of Family Medicine, National Taiwan University Hospital, Taipei 100225, Taiwan; 2 Department of Laboratory Medicine, National Taiwan University Hospital, National Taiwan University College of Medicine, Taipei 100225, Taiwan; 3 Department of Environmental and Occupational Medicine, National Taiwan University Hospital, Taipei 100225, Taiwan; 4 Institute of Environmental and Occupational Health Sciences, College of Public Health, National Taiwan University, Taipei 106319, Taiwan; 5 Division of Cardiology, Department of Internal Medicine, National Taiwan University Hospital, Taipei 100225, Taiwan

**Keywords:** glycative stress, HbA1c, atherogenic dyslipidemia, advanced glycation end-products

## Abstract

(1) Background: Diabetes mellitus (DM) is a significant health problem and is associated with dyslipidemia; however, the association between glycative stress, in terms of glycated hemoglobin (HbA1c), and atherogenic dyslipidemia in hyperlipidemic patients with and without DM has rarely been reported. (2) Methods: We prospectively recruited 949 hyperlipidemic patients from the Lipid Clinic of the National Taiwan University Hospital. HbA1c and fasting serum lipids, including total cholesterol (TC), high- and low-density lipoprotein cholesterol (HDL-C and LDL-C), small dense LDL-C (sdLDL-C), very low-density lipoprotein cholesterol (VLDL-C), triglycerides, and advanced glycation end-products (AGEs), were measured. After fasting for 10–14 h, all subjects except those with DM underwent a standard oral glucose tolerance test (OGTT) with 75 g of glucose loading. All subjects were asked to discontinue the use of lipid-lowering agents for 8 weeks before recruitment. (3) Results: Patients with DM had a higher prevalence of hypertension and higher levels of triglyceride, TC/HDL-C ratio, AGEs, VLDL-C, and sdLDL-C. Among patients with higher HbA1c, the serum VLDL-C, AGEs, and TC/HDL-C ratio were significantly higher than those with lower HbA1c. After adjustment for covariates, multiple logistic regression analyses revealed different groups of dysglycemia with higher HbA1c had a higher odds ratio for TC/HDL-C ≥ 5, sdLDL-C ≥ 75th percentile, VLDL-C ≥ 75th percentile and AGEs ≥ 75th percentile. (4) Conclusions: A higher HbA1c was associated with a significant increase in the risk of atherogenic dyslipidemia and AGEs levels in patients with hyperlipidemia. The findings can be very promising in clinical application.

## 1. Introduction

Diabetes mellitus (DM) poses a great global health threat and is estimated to affect 537 million adults worldwide [1]. In Taiwan, the total population and the prevalence of type II DM have both increased in all age groups in the past few years. According to the National Health Insurance Research Database, the number of residents aged 20–79 years old who were diagnosed with diabetes was approximately 16,486,000 in 2005, with a prevalence of 7.15%. Approximately 10 years later, however, it reached 17,935,000 in 2014, with a prevalence of 10.10% [2]. Further care for the increasing number of diabetic patients to reduce cardiovascular complications remains a great issue.

In addition to acute illness, diabetes is associated with long-term micro- and macrovascular complications and leads to significant mortality. In Taiwan, diabetic patients experience a significant loss of life, which in 2014 was estimated to be 2.6 and 3.2 years in women and men, respectively, when diabetes was diagnosed at 40 years of age. In regard to the causes of death in patients with diabetes, heart disease, and cerebrovascular disease occupied second and third place, respectively, from 2005 to 2014, following only malignancies. Remarkably, during the past 10 years, the mortality rate of diabetic patients dying from heart disease has increased significantly in both males and females [3].

For the evaluation of a high cardiovascular mortality rate in patients with diabetes, diabetic dyslipidemia is considered the pivotal pathogenic risk factor. DM is thought to be associated with alterations in lipid profiles, which could provide a strong link between diabetes and cardiovascular risk. Glycated hemoglobin (HbA1c) is considered to be associated with multiple lipid profiles and thus plays a crucial role in atherogenic dyslipidemia, especially in patients with DM, while the underlying pathophysiology is not yet understood comprehensively, particularly in subjects with impaired glucose tolerance and normoglycemia [4]. On the other hand, glycated hemoglobin is an index of diabetic control and an important prognostic marker of all-cause and cardiovascular complications, even in nondiabetic subjects [5,6]. Nevertheless, there is still limited evidence that indicates the connection between glycative stress in terms of glycated hemoglobin and atherogenic dyslipidemia in subjects with hyperlipidemia. This study is intended to evaluate the aforementioned association.

## 2. Materials and Methods

### 2.1. Patients

From 2009 to 2011, 949 patients with hyperlipidemia from the Lipid Clinic of National Taiwan University Hospital (NTUH) were consecutively enrolled in this study (653 males and 296 females aged 19 to 70 years old). Subjects were recruited with hyperlipidemia, defined as serum cholesterol levels greater than 200 mg/dL or triglyceride levels greater than 200 mg/dL. However, subjects with secondary hyperlipidemia, such as thyroid disease, nephrotic syndrome, chronic kidney disease with creatinine levels ≥ 3 mg/dL, obstructive liver diseases, malignant diseases, and those who were pregnant or taking drugs known to influence lipid metabolism, were excluded. Before study recruitment, all participants were never users of lipid-lowering therapy or had to discontinue lipid-lowering agents for 8 weeks. Thus, patients with documented coronary heart disease or cerebrovascular disease, or peripheral vascular disease were excluded. All of the participants fasted for 10–14 h, and venous blood samples were collected in the morning for measurement of serum lipid profiles and other biochemical markers. The informed consent of the subjects was obtained before recruitment. The study was ethically approved by the institutional review boards at the NTUH.

### 2.2. Lipids, Lipoprotein, and OGTT Measurements

The concentrations of lipids, including total cholesterol, triglyceride, and high-density and low-density lipoprotein cholesterol (HDL-C and LDL-C), were analyzed enzymatically using kits from Denka Seiken (Tokyo, Japan). The analyses were conducted using a Toshiba FR-200 automatic chemistry analyzer (Tokyo, Japan). Serum HDL-C and LDL-C were measured directly using a homogeneous enzymatic method with a coefficient of variation (CV) of 2%.

The levels of total cholesterol were first measured by a commercial kit (Denka Seiken, Japan), and the low-density lipoprotein cholesterol (VLDL-C) levels were then determined using agarose gel electrophoresis (Sebia, Norcross, GA, USA) with a CV of 2% to obtain the percentage of total cholesterol. This electrophoresis method can separate and measure the major lipoprotein components found in serum, including chylomicrons, beta lipoproteins or LDL-C, prebeta lipoproteins or VLDL-C, and alpha lipoproteins or HDL-C. The analysis was performed by electrophoresis on pH 7.5 buffered agarose gels. The separated lipoproteins were stained with a lipid-specific Sudan black stain. This quantitative method is in suitable accordance with reference ultracentrifugation results and was found to be reliable and highly suitable for clinical use by a previous study [7,8].

After measuring fasting blood samples, all subjects underwent an oral glucose tolerance test (OGTT) with 75 g of glucose loading in accordance with the World Health Organization standard. Then, venous blood samples were taken every 30 min until two hours following OGTT, and the results were classified according to the American Diabetes Association criteria: those who have less than 140 mg/dL (7.78 mmol/L) glucose are classified as normal, 140–199 mg/dL (7.78–11.06 mmol/L) have impaired glucose tolerance (IGT), and those with greater than 200 mg/dL (11.11 mmol/L) are diabetic based on the results of these 2 h blood glucose levels [9]. The plasma glucose concentration was determined using Denka Seiken reagent kits (Tokyo, Japan) with the hexokinase method conducted on a Toshiba FR-120 automatic chemistry analyzer. The CV for plasma glucose is under 3%.

### 2.3. AGE Measurement

AGEs were measured using the turbidimetric immunoassay method (Hanson Hong Biomedical Co., Ltd., Taipei, Taiwan) using a Hitachi 7150 (Osaka, Japan) analyzer, and the within and between CV was 1.3% and 2.8%, respectively.

### 2.4. Cardiovascular Risk Factors

Blood pressure (BP) measurements were performed using a mercury sphygmomanometer in a standardized fashion. Two measurements were taken after 5 min of rest in the sitting position. Hypertension was defined as subjects with a hypertension history or systolic BP ≥ 140 mmHg or diastolic BP ≥ 90 mmHg. Subjects having systolic BP between 120 and 139 mmHg or diastolic BP between 80 and 89 mmHg were defined as prehypertension, and normal BP was defined as systolic BP < 120 mmHg and diastolic BP < 80 mmHg. Body mass index (BMI) was calculated as the subject’s body weight in kilograms over body height in meters squared. The BMI subgroups were classified into 3 groups following the guidelines of the Taiwan Department of Health: <24 kg/m^2^ (normal), 24–27 kg/m^2^ (overweight), and ≥27 kg/m^2^ (obese). Data on alcohol use and smoking were obtained for each subject from a self-reported structural questionnaire. In this study, drinking two or more alcoholic drinks per week was defined as habitual drinking.

### 2.5. Statistical Analyses

Continuous variables are expressed as the means ± SD, and categorical data are expressed as the percentages for basic characteristics and lipid distribution. Stratified analysis for cardiovascular risk factors and atherogenic dyslipidemia was based on glycative stress (HbA1c levels) in different dysglycemic groups: HbA1c ≥ 7% vs. <7% in DM, HbA1c ≥ 6% vs. <6% in IGT, and HbA1c ≥ 5.7% vs. <5.7% in the NGT group.

Multivariate logistic regression analyses in terms of odds ratio (O.R.) (95% confidence interval, CI) were used to investigate the associations between different glycative stress categories and atherogenic dyslipidemia in all participants with different dysglycemic groups after controlling associated covariates.

All analyses were performed using SAS 9.1 (SAS Institute Inc., Cary, NC, USA). A *p*-value < 0.05 was considered statistically significant.

## 3. Results

### 3.1. Characteristics of Study Participants and Their Associations with Sex

Of all 949 participants, males accounted for 653 (68.8%) and females for 296 (31.2%). The age of the male participants was significantly lower than that of the female participants (46.7 ± 9.9 years old vs. 50.8 ± 13.0 years old, *p* < 0.001). The male group, when compared with the female group, had a significantly higher BMI (25.7 ± 3.2 kg/m^2^ vs. 24.1 ± 4.3 kg/m^2^, *p* < 0.001), alcohol use (16.23% vs. 6.08%, *p* < 0.001), and smoking habits (23.43% vs. 2.70%, *p* < 0.001). Significantly lower dyslipidemic parameters of males than those of females included TC (229.4 ± 64.1 mg/dL vs. 249.0 ± 80.1 mg/dL, *p* < 0.001), triglycerides (161 (95% CI, 113–259) mg/dL vs. 143 (95% CI, 91–258) mg/dL, *p* = 0.024), and HDL-C (50.4 ± 15.0 mg/dL vs. 59.8 ± 19.3 mg/dL, *p* < 0.001). There were no significant differences between the male and female groups in regard to blood pressure, fasting glucose level, the prevalence of hypertension and DM, habits of regular exercise, other dyslipidemic parameters (LDL-C, VLDL-C, sdLDL-C), or AGEs. The basic characteristics of the participants categorized by sex are listed in Table 1.

### 3.2. The Association between Dyslipidemia and Groups of Dysglycemia

Participants who received OGTT were categorized into DM, OGTT DM, IGT, and NGT according to the test results. As presented in Table 2, age, BMI, SBP, DBP, and prevalence of hypertension were significantly higher in trends from the NGT toward DM groups. No significant differences in different gender groups were noted. Among dyslipidemic parameters, triglyceride, TC/HDL-C ratio, TC/HDL-C ratio ≥ 5, VLDL-C, and sdLDL-C/LDL-C were progressively higher in trends from NGT toward DM groups, as were AGEs and sdLDL-C. Meanwhile, they were significantly lower in HDL-C and higher in TC and LDL-C. No significance was found in regard to non-high-density lipoprotein cholesterol (NHDL-C) levels or personal habits, such as the prevalence of exercise, alcohol use, and smoking habits.

### 3.3. The Association between Atherogenic Dyslipidemia and HbA1c Levels

After the OGTT test, all patients could be categorized into DM, OGTT DM, IGT, or NGT groups. Further analysis was carried out with stratification of HbA1c levels in each group. The results are presented in Table 3.

In the DM group, participants with higher HbA1c levels (HbA1c ≥ 7.0%) were significantly younger (49.7 ± 10.4 years old vs. 53.8 ± 10.8 years old, *p* = 0.010) and had a higher BMI (27.6 ± 3.5 kg/m^2^ vs. 25.9 ± 3.7 kg/m^2^, *p* = 0.003) than those with lower levels (HbA1c < 7.0%). No significant difference in male percentage was observed (75.7% vs. 64.6%, *p* = 0.109) in regard to dyslipidemia. Comparing groups with higher (HbA1c ≥ 7.0%) and lower HbA1c levels (HbA1c < 7.0%), there were significant differences in the serum levels of triglyceride (500.7 ± 49.8 mg/dL vs. 283.4 ± 40.5 mg/dL, *p* < 0.001), VLDL-C (93.8 ± 5.8 mg/dL vs. 72.7 ± 4.7 mg/dL, *p* < 0.006), the percentage of TC/HDL-C ratio ≥ 5 (60.8% vs. 40.9%, *p* < 0.008), and the ratio of sdLDL-C/LDL-C (0.4 ± 0.03 vs. 0.3 ± 0.02, *p* < 0.004). No significance was noted in TC, HDL-C, LDL-C, AGEs, and sdLDL-C; or personal habits, such as exercise, alcohol use, and smoking.

In the IGT group, participants with HbA1c ≥ 6.0% were significantly older than those with HbA1c < 6.0% (54.2 ± 8.1 years old vs. 49.3 ± 9.3 years old, *p* = 0.007), and the male percentage was significantly lower (56.4% vs. 77.9%, *p* = 0.019). Unlike the results in the DM group, the BMI of the two subgroups was similar. No significance was found in any dyslipidemic parameter or personal habits. Of note, a significant difference was found in AGEs (3.61 ± 0.65 arbitrary units vs. 5.22 ± 0.43 arbitrary units, *p* = 0.0462). 

In the NGT group, participants with HbA1c ≥ 5.7% were significantly older (48.5 ± 11.2 years old vs. 44.8 ± 10.8 years old, *p* < 0.001) and had a higher BMI and hypertension prevalence (31.12% vs. 24.38%, *p* = 0.048) than those with HbA1c < 5.7%. Higher HbA1c level was significantly associated with higher triglyceride level (243.8 ± 14.6 vs. 191.2 ± 11.2, *p* = 0.005), higher VLDL-C level (60.9 ± 2.6 mg/dL vs. 52.1 ± 2.0 mg/dL, *p* = 0.007), higher percentage of TC/HDL-C ratio ≥ 5 (36.5% vs. 21.4%, *p* < 0.001), higher sdLDL-C (37.5 ± 1.2 mg/dL vs. 34.1 ± 0.9 mg/dL, *p* = 0.026), and higher sdLDL-C/LDL-C ratio (0.3 ± 0.01 vs. 0.2 ± 0.01, *p* = 0.004). The AGE levels were similar between the subgroups. No significance was found in personal habits. Additional analysis with an HbA1c cutoff point of 5.5% was performed, and the results are shown in Table 4.

Taking the three groups together, higher glycative stress was significantly associated with older age, higher BMI, and higher hypertension percentage. Several atherogenic dyslipidemic parameters were associated with higher glycative stress, including higher levels of TC, triglycerides, LDL-C, and VLDL-C, and higher percentage of TC/HDL-C ratio ≥ 5. A higher glycative stress was positively associated with higher AGEs levels, sdLDL-C levels, sdLDL-C/LDL-C ratio, and lower HDL-C levels.

### 3.4. The Association between Atherogenic Dyslipidemia and Glycative Stress by Multivariate Regression Analyses

Multiple logistic regression analysis was carried out for the higher glycative stress and odds ratio of AGEs ≥ 75th percentile and atherogenic dyslipidemia, including TC/HDL-C ≥ 5, sdLDL-C ≥ 75th percentile, VLDL-C ≥ 75th percentile and sdLDL-C/LDL-C ≥ 75th percentile. Odds ratios were estimated with the subgroup of NGT whose HbA1c < 5.7% was the reference group. The results are presented in Table 5. After adjustment for covariates, patients with DM whose HbA1c level ≥ 7% faced the highest odds ratio among all groups in all five parameters (AGE ≥ 75th percentile: 4.11, 1.81–9.31; TC/HDL-C ≥ 5: 3.78, 2.16–6.59; sdLDL-C ≥ 75th percentile: 2.29, 1.31–4.03; sdLDL-C/LDL-C ≥ 75th percentile: 4.66, 2.64–8.22; VLDL-C ≥ 75th percentile: 4.76, 2.71–8.37). As with the DM group, the aforementioned dyslipidemic parameters in the IGT group also exhibited a higher trend associated with higher HbA1c, although only the association between TC/HDL-C ≥ 5 and HbA1c showed significance (2.16, 1.05–4.44). In the NGT group, all parameters except for AGE ≥ 75th percentile demonstrated significant associations with HbA1c level (TC/HDL-C ≥ 5: 1.91, 1.31–2.77; sdLDL-C ≥ 75th percentile: 1.62, 1.09–2.41; VLDL-C ≥ 75th percentile: 1.93, 1.28–2.90; sdLDL-C/LDL-C ≥ 75th percentile: 1.57, 1.04–2.39), indicating that higher glycative stress is associated with higher atherogenic dyslipidemia.

Significant associations between other variates and atherogenic dyslipidemia were also observed. Smoking, a notorious risk factor for cardiovascular disease, was associated with AGE ≥ 75th percentile, TC/HDL-C ≥ 5, VLDL-C ≥ 75th percentile and sdLDL-C/LDL-C ≥ 75th percentile. In contrast, alcohol was only significantly associated with sdLDL-C/LDL-C ≥ 75th percentile, while the odds ratio was higher than that for smoking (2.39, 1.55–3.68 vs. 1.71, 1.13–2.60). Hypertension was associated with TC/HDL-C ≥ 5, sdLDL-C/LDL-C ≥ 75th percentile, and VLDL-C ≥ 75th percentile. Notably, exercise was associated with lower TC/HDL-C ≥ 5 and sdLDL-C/LDL-C ≥ 75th percentile, suggesting protective characteristics against ASCVD risks.

Additional analysis with an HbA1c cutoff point of 5.5% was performed, and the results are shown in Table 6.

## 4. Discussion

A previous study in Korea raised the importance of glycated hemoglobin as a predictor for cardiometabolic risk in nondiabetic women, which demonstrated better performance than fasting glucose [10]. However, related evidence derived from large-scale research in Taiwan is scarce. Our study reveals that glycative stress by HbA1c value is strongly associated with several atherogenic dyslipidemia and AGEs in patients with hyperlipidemia, even in NGT and IGT groups, which have been validated by predicting cardiovascular outcomes and associated with cardiovascular risk factors. Consequently, the diagnosis of diabetes by clinical history or OGTT was also associated with atherogenic dyslipidemia. Further analysis was carried out on the basis of the previous grouping as well as according to the different levels of HbA1c in each group. Significance was noted in several parameters, such as CHO/HDL-C ≥ 5, sdLDL-C, VLDL-C, and sdLDL-C/LDL-C, regardless of the DM or NGT group. Additional sensitivity analysis of the NGT groups with different cutoff points of HbA1c, 5.5% and 5.7%, corroborated the study findings of significant associations between glycative stress and atherogenic dyslipidemia.

Due to the established evidence of the association between glycated hemoglobin and microvascular disease [11], the use of glycated hemoglobin has been recommended in the diagnosis and follow-up of diabetes. Glycated hemoglobin values could reflect 2-to-3 months of average endogenous exposure to glucose, and no fasting period is necessary immediately before measurement, which supports its clinical usefulness. Previous literature has documented the association between glycated hemoglobin and cardiovascular disease in nondiabetic patients [12], although the causal role of serum glucose itself remains unknown. On the other hand, the importance of diabetic dyslipidemia is increasingly highlighted, which may explain the relationship between glycated hemoglobin and cardiovascular disease [4,13,14,15]. The results of this study suggest the possible application of HbA1c in the evaluation and prediction of atherogenic dyslipidemia and cardiovascular risks.

We adopted several exclusion criteria in our study to eliminate confounders. CKD is associated with dyslipidemia, largely due to increased triglyceride levels, decreased HDL-C, and varying levels of LDL-C, attributed to complicated metabolic interference [16]. Usually, CKD is defined as an estimated glomerular filtration rate < 60 mL/min, which could be derived from the simplified modification of diet in renal disease (MDRD) equation. In our study, CKD patients with creatinine levels≥ 3 mg/dL were excluded to minimize the confounding factors. Likewise, patients treated with agents known to influence lipid metabolisms, such as steroids or insulin, were excluded.

Among all lipid profiles, LDL-C has been recognized as one of the most important indicators of atherosclerosis and cardiovascular disease. Small dense LDL-C, a subfraction of LDL-C, is particularly atherogenic, which is attributed to its lower affinity for the LDL receptor, higher penetration into the arterial wall, greater arterial retention due to increased binding to proteoglycans, and greater susceptibility to oxidation. SdLDL-C is associated with incident coronary heart disease and atherosclerotic risk markers, such as inflammation, thrombosis, hematological markers, and prediabetes, which supports its use as a promising biomarker of cardiovascular risks [17,18,19,20]. Another powerful predictor for the development of coronary heart disease in the Chinese population, the TC/HDL-C ratio with a cutoff point of 5, has superior specificity and accuracy and similar sensitivity when compared with LDL-C levels [21]. Our study reveals that different dysglycemic groups are closely associated with sdLDL-C and the percentage of TC/HDL-C ratio ≥ 5. Particularly, in the DM group, both sdLDL-C and the percentage of TC/HDL-C ratio ≥ 5 are strongly associated with a higher HbA1c level, suggesting its representative role. We also observed, in Table 2, that patients with DM live with lower LDL in contrast to significantly higher VLDL and TG. Previous evidence has shown that the complicated conversion of variable lipid profiles and lipid metabolism with LDL, VLDL, and sdLDL all play important roles in atherosclerosis [22]. The results indicate that LDL alone may not be representative enough when it comes to atherosclerosis.

The association between higher TG levels and ASCVD risks was once doubted. However, after adjusting for non-HDL-C, the association between plasma TG levels and ASCVD risks became nonsignificant, suggesting that the effect of plasma TGs on ASCVD is mediated by TG-rich lipoproteins as estimated by non-HDL-C, including TG-rich particles in VLDL-C and their remnants. TG-rich VLDL-C particles and their remnants are responsible for TG circulation, and a constant cholesterol/TG ratio in VLDL-C is assumed, which could be used to estimate VLDL-C levels. As one category of atherogenic ApoB-containing lipoproteins, VLDL-C participates in the mechanism of atherosclerosis [23,24]. In our study, VLDL-C was significantly associated with different hyperglycemic groups and different HbA1c levels in subgroups.

AGEs, a large family of protein, peptide, amino acid, nucleic acid, and lipid adducts formed by the reaction of carbonyl compounds derived directly or indirectly from glucose, ascorbic acid, and other metabolites, are considered to play important roles in diabetes-related complications. The link between AGEs and diabetic micro- and macrovascular complications has been observed. They are also involved in coronary artery disease pathogenesis by crosslinking extracellular matrix proteins, increasing vascular stiffness, and disrupting endothelial homeostasis [25,26,27]. In our study, an important glycative stress marker of AGE was measured, and a significant trend was observed among the different dysglycemic groups. Furthermore, the odds ratio of AGEs ≥ 75th percentile was significantly associated with higher HbA1c levels in the DM groups. This result is compatible with previously known mechanisms of AGE formation in patients with diabetes.

Although the actual relationship has not yet been established, the fact that the HbA1c value is associated with cardiovascular risk, as well as atherogenic dyslipidemia, could be confirmed, and the association with atherogenic dyslipidemia could be potentially pathogenic. Atherogenic dyslipidemia should be given more attention, particularly in subjects with higher glycated hemoglobin levels independent of diabetes, new-onset diabetes, nondiabetes, IGT, and normal glucose levels. On the other hand, for patients with known atherogenic dyslipidemia or other cardiovascular risks, glycated hemoglobin might be considered an important risk marker. How HbA1c influences atherogenic dyslipidemia, how it contributes to cardiovascular risk, and how it will direct clinical intervention remain issues that require further study.

There are several nonnegligible limitations in this study. First, our study is based on a cross-sectional design, and thus, a causal role could not be identified, although the association between HbA1c and atherogenic dyslipidemia was established. Second, we consecutively recruit patients from the outpatient clinic for dyslipidemia, and unbalanced gender distribution is noted. Since we adopted several exclusion criteria, our results could not be generalized to other patient groups. In addition, we included participants who were never users of lipid-lowering therapy or had discontinued lipid-lowering agents for 8 weeks. Previous studies have demonstrated a rebound phenomenon of inflammatory response after statin withdrawal within days, especially in patients with acute CHD [28,29,30,31,32]. The changes in inflammatory stress may contribute to the adverse cardiovascular outcome of patients. However, whether the pharmacological effects diminish enough and lipid levels regress to each patient’s baseline remains a concern. Finally, the case number in the IGT group was relatively low, which might explain why the significance of several parameters in the IGT groups could not be identified. Further study with a larger case number is needed to analyze the association.

## 5. Conclusions

In conclusion, glycative stress by HbA1c is strongly associated with atherogenic dyslipidemia in patients with hyperlipidemia, which might be the pathogenic and mediating factors of higher cardiovascular complications in different categories of dysglycemia, such as DM, OGTT DM, and hyperglycemia or IGT, or even in subjects of normoglycemia. The clinical usefulness of HbA1c as a marker of atherogenic dyslipidemia needs more evidence from population-based studies, and more large-scale and longitudinal studies are warranted. 

## Figures and Tables

**Table 1:** Baseline characteristics of the study participants.

	Male (n = 653)	Female (n = 296)	*p*-Value
Age, years	46.7 ± 9.9	50.8 ± 13.0	<0.001
Body mass index, kg/m^2^	25.7 ± 3.2	24.1 ± 4.3	<0.001
SBP, mmHg	124.6 ± 14.3	125.9 ± 17.2	0.265
DBP, mmHg	77.2 ± 9.9	76.5 ± 10.6	0.323
Hypertension, %	32.92	37.84	0.140
Fasting glucose, mg/dL	101.0 ± 29.5	98.0 ± 24.1	0.099
Diabetes mellitus, %	150(22.97)	59(19.93)	0.295
Total cholesterol, mg/dL	229.4 ± 64.1	249.0 ± 80.1	<0.001
Triglycerides, mg/dL	161 (113–259)	143 (91–258)	0.024
HDL-C, mg/dL	50.4 ± 15.0	59.8 ± 19.3	<0.001
LDL-C, mg/dL	136.3 ± 52.3	137.1 ± 62.1	0.842
VLDL-C, mg/dL	62.6 ± 40.0	59.2 ± 49.5	0.313
sdLDL-C, mg/dL	36.5 ± 17.8	38.0 ± 22.3	0.289
AGEs, arbitrary units	4.5 ± 2.6	4.6 ± 3.0	0.747
Exercise, %	22.51	20.61	0.511
Current alcohol, %	16.23	6.08	<0.001
Current smoking, %	23.43	2.70	<0.001

Abbreviations: SBP, systolic blood pressure; DBP, diastolic blood pressure; HDL-C, high-density lipoprotein cholesterol; LDL-C, low-density lipoprotein cholesterol; VLDL-C, very low-density lipoprotein cholesterol; sdLDL-C, small dense low-density lipoprotein cholesterol; AGEs, advanced glycation end-products.

**Table 2:** Cardiovascular factors in clinical subjects receiving OGTT.

	DM (n = 144)	OGTT DM (n = 42)	IGT (n = 109)	NGT (n = 654)	*p*-Value for Trend
Male, %	64.6	85.7	69.7	68.5	0.076
Age, years	52.6 ± 11.5	50.8 ± 7.8	51.1 ± 9.1	46.3 ± 11.1	<0.001
BMI, kg/m^2^	26.8 ± 3.7	25.7 ± 3.5	25.4 ± 3.2	24.8 ± 3.6	<0.001
SBP ^a^, mmHg	128.2 ± 1.2	128.6 ± 2.2	125.4 ± 1.4	124.0 ± 0.6	0.005
DBP ^a^, mmHg	79.9 ± 0.8	80.2 ± 1.5	77.7 ± 0.9	76.0 ± 0.4	<0.001
Hypertension, %	54.2	50.0	46.8	27.1	<0.001
TC ^a^	233.6 ± 6.0	234.6 ± 10.7	214.7 ± 6.7	239.5 ± 2.8	0.009
Triglyceride ^a^	369.5 ± 23.9	318.4 ± 43.0	254.9 ± 26.8	215.1 ± 11.0	<0.001
HDL-C ^a^	46.8 ± 1.4	52.4 ± 2.4	47.9 ± 1.5	55.8 ± 0.6	<0.001
LDL-C ^a^	120.6 ± 4.7	132.3 ± 8.5	122.2 ± 5.3	142.8 ± 2.2	<0.001
NHDL-C ^a^	186.8 ± 5.5	182.1 ± 9.8	166.8 ± 6.1	183.7 ± 2.5	0.060
TC/HDL-C ^a^	5.2 ± 0.1	4.8 ± 0.2	4.8 ± 0.1	4.5 ± 0.1	<0.001
TC/HDL-C ≥ 5, %	52.1	40.5	37.6	27.7	<0.001
VLDL-C ^a^	78.7 ± 3.6	75.8 ± 6.4	62.8 ± 4.0	56.6 ± 1.6	<0.001
Current alcohol, %	16.23	6.08			<0.001
Current smoking, %	23.43	2.70			<0.001

^a^ adjusted for age and BMI. Abbreviations: OGTT, oral glucose tolerance test; DM, diabetes mellitus; IGT, impaired glucose tolerance; NGT, normal glucose tolerance; BMI, body mass index; SBP, systolic blood pressure; DBP, diastolic blood pressure; TC, total cholesterol; HDL-C, high-density lipoprotein cholesterol; LDL-C, low-density lipoprotein cholesterol; NHDL-C, non-high-density lipoprotein cholesterol; VLDL-C, very low-density lipoprotein cholesterol; AGEs, advanced glycation end-products; sdLDL-C, small dense low-density lipoprotein cholesterol.

**Table 3:** Cardiovascular characteristics of clinical subjects after OGTT stratified by glycated hemoglobin levels.

	DM			IGT			NGT			
HbA1c (%)	≥7.0 (n = 75)	<7.0 (n = 111)	*p*1-Value	≥6.0 (n = 40)	<6.0 (n = 69)	*p*2-Value	≥5.7 (n = 244)	<5.7 (n = 410)	*p*3-Value	*p*4-Value
Male, %	75.7	64.6	0.109	56.4	77.9	0.019	68.5	68.5	0.998	0.942
Age, yr	49.7 ± 10.4	53.8 ± 10.8	0.010	54.2 ± 8.1	49.3 ± 9.3	0.007	48.5 ± 11.2	44.8 ± 11	<0.001	<0.001
BMI, kg/m^2^	27.6 ± 3.5	25.9 ± 3.7	0.003	25.4 ± 3.2	25.5 ± 3.3	0.908	25.6 ± 4.1	24.3 ± 3.2	<0.001	<0.001
HTN, %	47.30	57.27	0.104	48.72	47.06	0.827	31.12	24.38	0.048	<0.001
TC ^a^	245.8 ± 10.9	231.4 ± 8.9	0.316	212.2 ± 8	219.7 ± 6	0.461	241 ± 4.3	236 ± 3.3	0.329	0.012
Triglyceride ^a^	500.7 ± 49.8	283.4 ± 40.5	0.001	264.6 ± 40.9	247.6 ± 30.7	0.744	243.8 ± 14.6	191.2 ± 11.2	0.005	<0.001
HDL-C ^a^	44.3 ± 2.0	48.5 ± 1.6	0.110	47.3 ± 2.4	48.4 ± 1.8	0.723	55.1 ± 1.0	56.9 ± 0.8	0.194	<0.001
LDL-C ^a^	117.3 ± 7.6	130.4 ± 6.2	0.190	117.9 ± 6.8	126.3 ± 5.1	0.338	140.9 ± 3.5	142.4 ± 2.7	0.727	<0.001
VLDL-C ^a^	93.8 ± 5.8	72.7 ± 4.7	0.006	64.9 ± 6.4	62.0 ± 4.8	0.725	60.9 ± 2.6	52.1 ± 2.0	0.007	<0.001
TC/HDL-C ≥ 5, %	60.8	40.9	0.008	41.0	35.3	0.555	36.5	21.4	<0.001	<0.001
AGEs ^a^	5.85 ± 0.47	5.16 ± 0.37	0.265	3.61 ± 0.65	5.22 ± 0.43	0.046	4.52 ± 0.21	4.19 ± 0.15	0.213	0.008
sdLDL-C ^a^	43.8 ± 2.6	41.9 ± 2.1	0.580	35.7 ± 2.8	35.8 ± 2.1	0.979	37.5 ± 1.2	34.1 ± 0.9	0.026	0.020
sdLDL-C /LDL-C ^a^	0.4 ± 0.03	0.3 ± 0.02	0.004	0.3 ± 0.02	0.3 ± 0.02	0.649	0.3 ± 0.01	0.2 ± 0.01	0.004	<0.001
Exercise, %	22.97	20.91	0.739	25.64	20.59	0.547	19.09	23.89	0.155	0.990
Alcohol, %	17.57	15.45	0.704	2.56	14.71	0.053	11.20	12.56	0.608	0.227
Smoking, %	28.38	17.27	0.073	23.08	22.06	0.903	11.62	16.26	0.106	0.018

^a^ adjusted for age and BMI. Abbreviations: OGTT, oral glucose tolerance test; DM, diabetes mellitus; IGT, impaired glucose tolerance; NGT, normal glucose tolerance. BMI, body mass index; HTN, hypertension; TC, total cholesterol; HDL-C, high-density lipoprotein cholesterol; LDL-C, low-density lipoprotein cholesterol; VLDL-C, very low-density lipoprotein cholesterol; AGEs, advanced glycation end-products; sdLDL-C, small dense low-density lipoprotein cholesterol. Unit of lipids: mg/dL, unit of AGEs: arbitrary units.

**Table 4:** Cardiovascular characteristics of clinical subjects after OGTT stratified by glycation hemoglobin levels, with a cutoff point of 5.5% in the NGT group.

	DM			IGT			NGT			
HbA1c (%)	≥7.0 (n = 75)	<7.0 (n = 111)	*p*1-Value	≥6.0 (n = 40)	<6.0 (n = 69)	*p*2-Value	≥5.5% (n = 408)	<5.5% (n = 246)	*p*3-Value	*p*4-Value
TC	245.8 ± 10.9	231.4 ± 8.9	0.316	212.2 ± 8.0	219.7 ± 6.0	0.461	240.4 ± 3.3	233.3 ± 4.3	0.195	0.012
Age, yr	49.7 ± 10.4	53.8 ± 10.8	0.010	54.2 ± 8.1	49.3 ± 9.3	0.007	48.5 ± 11.2	44.8 ± 11	<0.001	<0.001
BMI, kg/m^2^	27.6 ± 3.5	25.9 ± 3.7	0.003	25.4 ± 3.2	25.5 ± 3.3	0.908	25.6 ± 4.1	24.3 ± 3.2	<0.001	<0.001
HTN, %	47.30	57.27	0.104	48.72	47.06	0.827	31.12	24.38	0.048	<0.001
TC ^a^	245.8 ± 10.9	231.4 ± 8.9	0.316	212.2 ± 8	219.7 ± 6	0.461	241 ± 4.3	236 ± 3.3	0.329	0.012
Triglyceride ^a^	500.7 ± 49.8	283.4 ± 40.5	0.001	264.6 ± 40.9	247.6 ± 30.7	0.744	243.8 ± 14.6	191.2 ± 11.2	0.005	<0.001
HDL-C ^a^	44.3 ± 2.0	48.5 ± 1.6	0.110	47.3 ± 2.4	48.4 ± 1.8	0.723	55.1 ± 1.0	56.9 ± 0.8	0.194	<0.001
LDL-C ^a^	117.3 ± 7.6	130.4 ± 6.2	0.190	117.9 ± 6.8	126.3 ± 5.1	0.338	140.9 ± 3.5	142.4 ± 2.7	0.727	<0.001
VLDL-C ^a^	93.8 ± 5.8	72.7 ± 4.7	0.006	64.9 ± 6.4	62.0 ± 4.8	0.725	60.9 ± 2.6	52.1 ± 2.0	0.007	<0.001
TC/HDL-C ≥ 5, %	60.8	40.9	0.008	41.0	35.3	0.555	36.5	21.4	<0.001	<0.001
AGEs ^a^	5.85 ± 0.47	5.16 ± 0.37	0.265	3.61 ± 0.65	5.22 ± 0.43	0.046	4.52 ± 0.21	4.19 ± 0.15	0.213	0.008
sdLDL-C ^a^	43.8 ± 2.6	41.9 ± 2.1	0.580	35.7 ± 2.8	35.8 ± 2.1	0.979	37.5 ± 1.2	34.1 ± 0.9	0.026	0.020
sdLDL-C /LDL-C ^a^	0.4 ± 0.03	0.3 ± 0.02	0.004	0.3 ± 0.02	0.3 ± 0.02	0.649	0.3 ± 0.01	0.2 ± 0.01	0.004	<0.001

^a^ All lipid items are adjusted for age and BMI. Abbreviations: OGTT, oral glucose tolerance test; DM, diabetes mellitus; IGT, impaired glucose tolerance; NGT, normal glucose tolerance; TC, total cholesterol; HDL-C, high-density lipoprotein cholesterol; LDL-C, low-density lipoprotein cholesterol; NHDL-C, non-high-density lipoprotein cholesterol; VLDL-C, very low-density lipoprotein cholesterol; AGEs, advanced glycation end-products; sdLDL-C, small dense low-density lipoprotein cholesterol. Unit of lipids: mg/dL. Unit of AGEs: arbitrary units.

**Table 5:** Multivariate logistic regression model for the odds ratio of atherogenic dyslipidemia in all participants.

	AGEs ≥ 75th Percentile	TC/HDL-C ≥ 5	sdLDL-C ≥ 75th Percentile	sdLDL-C/LDL-C ≥ 75th Percentile	VLDL-C ≥ 75th Percentile
HbA1c (%)	O.R. (95% CI)	O.R. (95% CI)	O.R. (95% CI)	O.R. (95% CI)	O.R. (95% CI)
DM, ≥7%	4.11 (1.81–9.31 ) ‡	3.78 (2.16–6.59) ‡	2.29 (1.31–4.03) ‡	4.66 (2.64–8.22) ‡	4.76 (2.71–8.37) ‡
<7%	2.05 (1.02–4.13) *	1.93 (1.18–3.14) †	2.18 (1.32–3.59) ‡	2.04 (1.22–3.41) †	1.96 (1.16–3.29) *
IGT, ≥6%	0.79 (0.25–2.53)	2.16 (1.05–4.44) *	1.39 (0.63–3.08)	1.87 (0.86–4.10)	2.02 (0.94–4.35)
<6%	1.73 (0.85–3.53)	1.56 (0.87–2.78)	1.18 (0.62–2.26)	1.50 (0.80–2.82)	1.58 (0.84–2.98)
NGT, ≥5.7%	1.44 (0.88–2.37)	1.91 (1.31–2.77) ‡	1.62 (1.09–2.41) *	1.57 (1.04–2.39) *	1.93 (1.28–2.90) ‡
<5.7%	1	1	1	1	1
Male	0.48 (0.28–0.80) †	1.15 (0.81–1.63)	0.68 (0.48–0.98) *	0.76 (0.52–1.09)	0.69 (0.48–1.00)
Age	1.00 (0.97–1.02)	1.01 (0.99–1.02)	1.02 (1.00–1.03) *	1.01 (1.00–1.03)	1.00 (0.98–1.01)
BMI	1.09 (1.02–1.16) *	1.10 (1.06–1.16) ‡	1.09 (1.04–1.14) ‡	1.05 (1.00–1.10) *	1.05 (1.00–1.10) *
Smoking	1.68 (1.01–2.79) *	1.92 (1.31–2.83) ‡	1.10 (0.71–1.68)	1.71 (1.13–2.60) *	2.72 (1.82–4.07) ‡
Alcohol	1.78 (0.90–3.52)	1.32 (0.86–2.03)	1.42 (0.91–2.21)	2.39 (1.55–3.68) ‡	1.49 (0.95–2.34)
Hypertension	1.34 (0.84–2.16)	1.46 (1.06–2.00) *	0.93 (0.66–1.31)	1.42 (1.01–1.99) *	1.59 (1.14–2.24) †
Exercise	1.23 (0.77–1.98)	0.62 (0.42–0.91) *	0.89 (0.61–1.31)	0.60 (0.39–0.92) *	0.78 (0.52–1.18)

*p*-value: * <0.05, † <0.01, ‡ <0.005. Reference group was defined as normal glucose tolerance (NGT) with <5.7%. Abbreviations: TC, total cholesterol; HDL-C, high-density lipoprotein cholesterol; AGEs, advanced glycation end-products; sdLDL-C, small dense low-density lipoprotein cholesterol; VLDL-C, very low-density lipoprotein cholesterol; DM, diabetes mellitus; IGT, impaired glucose tolerance; NGT, normal glucose tolerance; BMI, body mass index.

**Table 6:** Multivariate logistic regression model for all participants, with a cutoff point of HbA1c ≥ 5.5% in the NGT group.

	AGEs ≥ 75th Percentile	TC/HDL-C ≥ 5	sdLDL-C ≥ 75th Percentile	VLDL-C ≥ 75th Percentile	NHDL-C ≥ 75th Percentile
HbA1c (%)	O.R. (95% CI)	O.R. (95% CI)	O.R. (95% CI)	O.R. (95% CI)	O.R. (95% CI)
DM, ≥7%	4.35 (1.83–10.35) ‡	3.98 (2.18–7.25) ‡	3.00 (1.60–5.63) ‡	5.68 (3.04–10.61) ‡	1.44 (0.77–2.68)
<7%	2.16 (1.02–4.60) *	2.03 (1.18–3.47) *	2.86 (1.62–5.07) ‡	2.33 (1.30–4.20) ‡	1.10 (0.62–1.95)
IGT, ≥6%	0.84 (0.25–2.76)	2.29 (1.07–4.87) *	1.83 (0.79–4.25)	2.43 (1.08–5.46) *	0.88 (0.37–2.09)
<6%	1.83 (0.85–3.92)	1.65 (0.89–3.08)	1.55 (0.77–3.13)	1.90 (0.96–3.76)	0.96 (0.49–1.91)
NGT, ≥5.5%	1.36 (0.81–2.26)	1.63 (1.09–2.43) *	1.97 (1.27–3.07) ‡	1.95 (1.25–3.07) ‡	1.46 (0.98–2.16)
< 5.5%	1	1	1	1	1
Male	0.47 (0.28–0.78) ‡	1.13 (0.79–1.60)	0.67 (0.47–0.95) *	0.68 (0.47–0.98) *	0.64 (0.45–0.90) *
Age	1.00 (0.97–1.02)	1.01 (0.99–1.02)	1.02 (1.00–1.03) *	0.99 (0.98–1.01)	1.01 (1.00–1.03)
BMI	1.09 (1.02–1.16) *	1.11 (1.06–1.16) ‡	1.09 (1.04–1.14) ‡	1.05 (1.01–1.10) *	1.03 (0.99–1.08)
Smoking	1.65 (0.99–2.73)	1.85 (1.26–2.72) ‡	1.08 (0.70–1.65)	2.64 (1.77–3.94) ‡	1.00 (0.65–1.53)
Alcohol	1.79 (0.90–3.53)	1.33 (0.87–2.04)	1.45 (0.93–2.27)	1.53 (0.98–2.39)	1.62 (1.05–2.50) *
Hypertension	1.35 (0.84–2.17)	1.44 (1.05–1.99) *	0.93 (0.66–1.30)	1.59 (1.13–2.23) †	0.96 (0.68–1.34)
Exercise	1.21 (0.76–1.95)	0.60 (0.41–0.89) *	0.89 (0.60–1.30)	0.77 (0.51–1.16)	0.80 (0.54–1.17)

*p*-value: * <0.05, † <0.01, ‡ <0.005. Abbreviations: TC, total cholesterol; HDL-C, high-density lipoprotein cholesterol; AGEs, advanced glycation end-products; sdLDL-C, small dense low-density lipoprotein cholesterol; VLDL-C, very low-density lipoprotein cholesterol; NHDL, non-high-density lipoprotein cholesterol; DM, diabetes mellitus; IGT, impaired glucose tolerance; NGT, normal glucose tolerance; BMI, body mass index.

## Data Availability

All relevant data are within the manuscript.
